# QuickStats

**Published:** 2013-11-22

**Authors:** Jiaquan Xu

**Figure:**
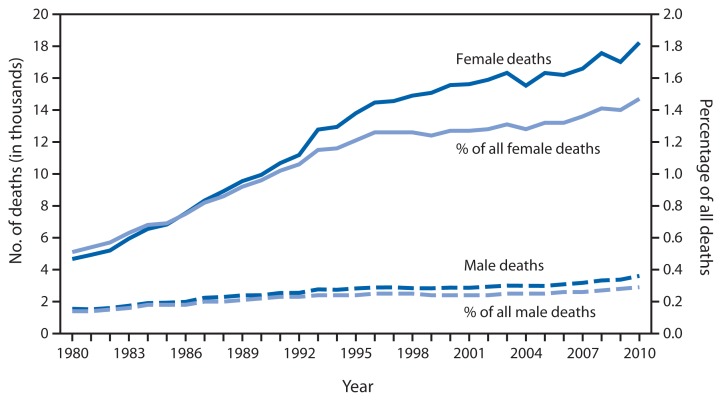
Number of Deaths Among Centenarians and Percentage Among All Deaths, by Sex — United States, 1980–2010

As more persons in the United States reach the age of 100 years, the number of deaths of those aged ≥100 years has been increasing. From 1980 to 2010, the number of deaths among female centenarians increased from 4,668 to 18,222, and the number of deaths among male centenarians increased from 1,552 to 3,607. Throughout the period, the number of deaths among female centenarians ranged from three to five times higher than the number among males. The percentage of centenarian deaths among all deaths also increased, from 0.51% to 1.47% among females and from 0.14% to 0.29% among males.

**Source:** National Vital Statistics System. Mortality public use data files, 1980–2010. Available at http://www.cdc.gov/nchs/data_access/vitalstatsonline.htm.

